# Impacts of Skin Color and Hypoxemia on Noninvasive Assessment of Peripheral Blood Oxygen Saturation: A Scoping Review

**DOI:** 10.7759/cureus.46078

**Published:** 2023-09-27

**Authors:** Kawaiola C Aoki, Maya Barrant, Mam Jarra Gai, Marina Handal, Vivian Xu, Harvey N Mayrovitz

**Affiliations:** 1 Medical School, Nova Southeastern University Dr. Kiran C. Patel College of Osteopathic Medicine, Fort Lauderdale, USA; 2 Medical Education and Simulation, Cardiopulmonary Physiology, Nova Southeastern University Dr. Kiran C. Patel College of Allopathic Medicine, Fort Lauderdale, USA

**Keywords:** racial disparity, racial bias, skin color, occult hypoxia, electronic monitoring devices, false hypoxemia, skin pigmentation, pulse oximetry

## Abstract

Standard pulse oximeters estimate arterial blood saturation (SaO_2_) non-invasively by emitting and detecting light of a specific wavelength through a cutaneous vascular bed, such as a digit or the ear lobe. The quantity measured at these peripheral sites is designated as oxygen saturation (SpO_2_). Most reliable pulse oximeters are calibrated from measurements of healthy volunteers using some form of oxygen desaturation method. As the degree of inducible hypoxemia is limited, the calibration below achievable desaturation levels is usually extrapolated, leading to potential measurement error at low SaO_2_ values, especially in highly pigmented skin. Such skin color-related errors (SCRE) are the topic of this scoping review. Specifically, this study aimed to identify the combined impact of skin color and reduced SaO_2_ on the non-invasive assessment of SpO_2 _and report the consequences of potential inaccuracies.

Three databases were searched (Cumulated Index to Nursing and Allied Health Literature (CINAHL), PubMed, and Web of Science) for peer-reviewed prospective and retrospective studies published in English between 2000 and 2022 involving human patients with hypoxemia that included a measure of skin color (Fitzpatrick scale or race/ethnicity). Ten studies met the criteria and were included in the final review. Eight of these studies reported statistically significant higher pulse oximeter readings in darker-skinned patients with hypoxia compared to their arterial blood gas measurements. Occult hypoxia was more prevalent in Black and Hispanic patients than in White patients. Minority patients overall (Black, Asian, and American Indian) were more likely to have a SaO_2_ < 88% that was not detected by pulse oximetry (occult hypoxemia) during hospitalization. With greater levels of hypoxemia, the differences between SpO_2_ and SaO_2 _were greater. If SaO_2_ was < 90%, then SpO_2_ was overestimated in all ethnicities but worse in minorities. In conclusion, the bias found in pulse oximeter readings in the skin of color broadly impacts patients with hypoxemia. The failure of SpO_2_ measuring devices to detect occult hypoxemia can delay the delivery of life-saving treatment to critically ill patients requiring respiratory rehabilitation and supplemental oxygen therapy. This may lead to adverse health outcomes, increased in-hospital mortality, and complications such as organ dysfunction. An improvement in pulse oximeter detection mechanisms that would include all skin pigmentations is therefore much desired to optimize individual healthcare status and minimize disparities in treatment.

## Introduction and background

Standard pulse oximeters estimate arterial blood saturation (SaO_2_) noninvasively by emitting and detecting light of a specific wavelength through a cutaneous vascular bed, such as a digit or the ear lobe. The quantity measured at these peripheral sites is designated as oxygen saturation (SpO_2_). The SpO_2_ measurement by the devices uses light-emitting diodes (LEDs) at separate wavelengths, usually centered at a red wavelength of 660 nm and an infrared wavelength centered at about 940 nm. Using these two wavelengths makes distinguishing between oxy- and deoxyhemoglobin possible. A photodiode detector opposing the light source (transmissive mode) or adjacent to the light source (reflective mode) can detect the intensity of transmitted light through the tissue, or reflected from it, at each wavelength to derive the O_2_ saturation denoted as SpO_2_ [1,2]. However, the absorbance of light by the photodiode can be interrupted by other obstacles, causing scatter, reflection, and light absorbance by other tissue and blood components. Consequently, the pulse oximeter must isolate the absorbance of arterial blood from other parts detected. When this is done, the ratios of reduced to oxygenated hemoglobin are detected via the use of dual wavelengths and converted to SpO_2_ saturation [1,3].

On occasion, even within the pulsatile fraction of arterial blood, the effects of reflection and scattering of light may cause an overestimation of SpO_2_ [4]. This overestimation of true SaO_2_ appears to increase with decreasing SpO_2_ values [5,6], especially for SpO_2_ values <90%. This increases errors between SpO_2_ and true SaO_2_ [7,8]. Several studies support a linear decrease in SpO_2_ values in hypoxic patients, with differences attributed to the brand of the pulse oximeter, even when there is a good correlation between in vitro oximeter and pulse oximeter readings [8,9]. In patients with low SpO_2_ values and poor perfusion, finger probes are reported to be more accurate than nose or forehead probes [8].

Skin pigmentation, or, as some refer to it, skin tone or skin color, is a factor that may affect measured SpO_2_ values [9,10], with dark pigmentation reported to cause detection errors [11]. This is in part related to the dependence of the detected light intensity on melanin absorption patterns and wavelengths [12,13]. Furthermore, other studies reported that SpO_2_ values determined by pulse oximetry overestimate true SaO_2_ values in persons with darkly pigmentated skin [14,15], although some reports indicate either overestimation or underestimation in persons of color [16,17]. Information on the accuracy of commercial and medical-grade pulse oximeters is scarce, especially as it may be affected by skin tone, and it is not always clear if a suitably diverse population was used to test the accuracy or calibrate the algorithms subsequently used in the commercially available devices. Examples of potentially important application errors include studies that indicate increased SpO_2_ error with decreasing SaO_2_ [5,6]. In this scoping review, we sought to identify and summarize the compounded effects of an intrinsic potential inaccuracy of SpO_2_ readings in persons with darker skin pigmentation and hypoxemia (SaO_2_ ≤90%). The goal was to characterize and emphasize the need to consider including diverse skin types when developing such devices.

## Review

Methods

This scoping review was done using the procedures described by Arksey and O'Malley [18] and Levac et al. [19]. The review was executed in the following five phases: identifying the research question, identifying relevant studies, study selection, charting the data, and collating, summarizing, and reporting the results.

Eligibility Criteria

Original peer-reviewed articles published in English between 2000 and 2023 involving human studies were eligible for inclusion. Prospective and retrospective studies were included; case studies and reviews were excluded. To be included, the studies must also involve patients with hypoxemia (SaO_2_ ≤ 90% or International Classification of Diseases, Tenth Revision (ICD-10) code R09.02) and a measure of skin color (such as the Fitzpatrick scale or indirectly via race or ethnicity). None of the final studies had Fitzpatrick. The Preferred Reporting Items for Systematic Reviews and Meta-Analyses (PRISMA) flowchart [20] was used to organize the inclusion process systematically.

Information Sources

Electronic databases Cumulated Index to Nursing and Allied Health Literature (CINAHL) (EBSCOhost), PubMed, and Web of Science were accessed. PubMed is a free search engine open to the public with access to the Medical Literature Analysis and Retrieval System Online (MEDLINE) database. The CINHAL is a database housing nursing, biomedicine, and healthcare publications. The Web of Science is a search engine with access to multiple databases across many academic disciplines.

Search Strategy

**Figure 1:**
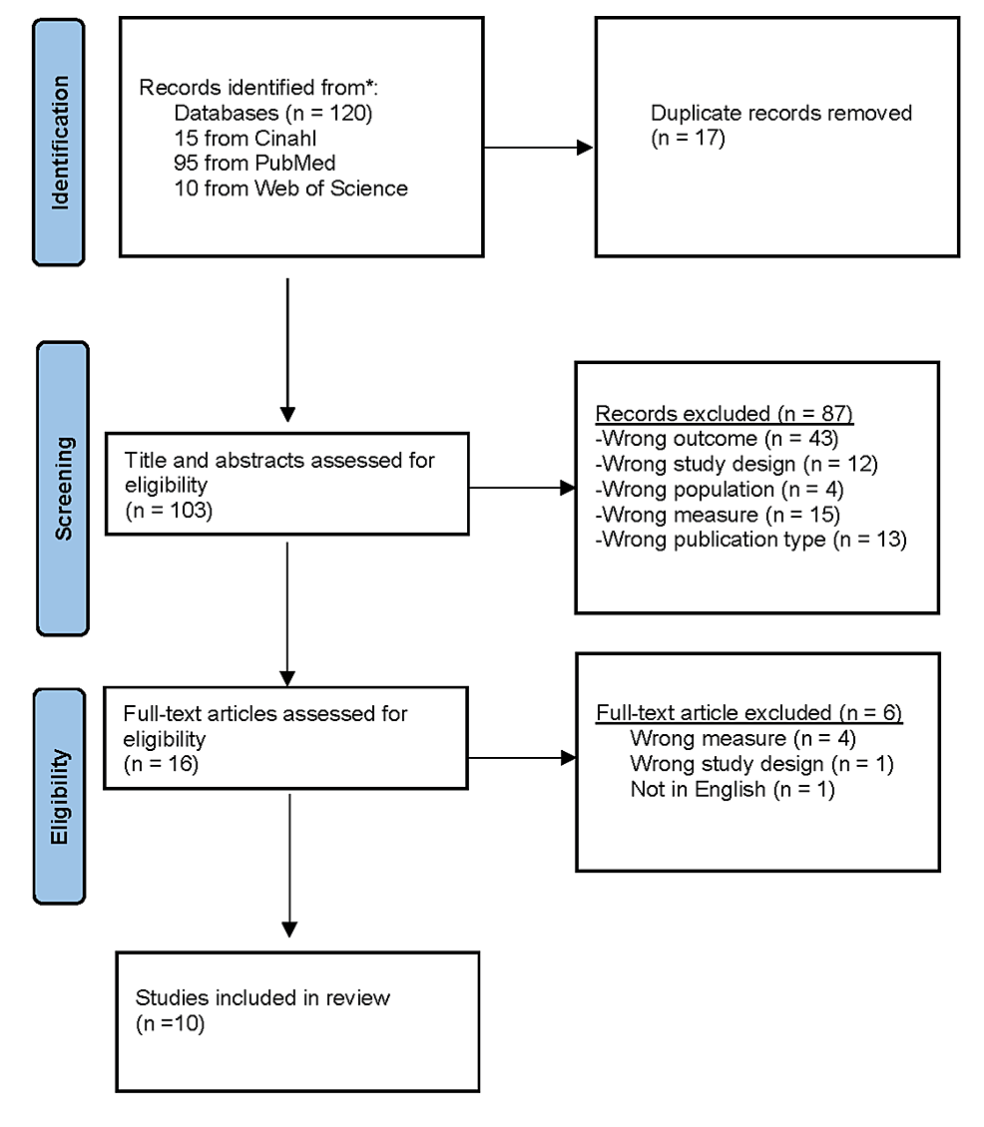
The PRISMA flowchart outlines the process of study selection The Preferred Reporting Items for Systematic Reviews and Meta-Analyses (PRISMA) flow diagram shows the literature search, screening, and final inclusion of studies. CINAHL: Cumulated Index to Nursing and Allied Health Literature

Two reviewers conducted initial, independent searches. In this review, we included publications that examined skin color and pigmentation and their effects on pulse oximetry. The search terms 'skin,' 'pulse oximetry,' 'pulse oximeter,' 'pulse ox,' 'hypoxia,' 'hypoxic,' 'hypoxemic,' and 'hypoxemia' were entered into the controlled descriptors for CINAHL, PubMed, and Web of Science. The Boolean operator AND was used for simultaneous occurrences and OR for their synonyms. The search terms used can be seen in the PRISMA flow diagram (Figure 1).

All identified search articles were transferred to an online systematic review software (Rayyan, Qatar Computing Research Institute, Doha, Qatar).

*Study Selection Process* 

The search identified 125 publications, and after 18 duplicates were removed, there remained 107 distinct articles to be further analyzed for relevance. All reviewers performed initial screening on every title and abstract. Publications identified that met initial screening inclusion criteria were discussed to determine if the full-text article was likely relevant and should be retained for further analysis. Discrepancies between reviewers on the initial screening were resolved by consensus, and 10 studies were retained for further analysis.

Quality Assessment

The quality assessment of the final articles was completed using the Joanna Briggs Institute Critical Appraisal Tools [21]. These tools allow for methodological evaluation of the study design and the risk of bias according to study type. Two reviewers individually appraised the included articles using the appropriate tool and then reconvened and compared the results until a consensus was reached. Using these tools, the included articles were classified according to their risk of bias: high risk (<50%), moderate risk (50%-70%), and low risk (>70%).

Data Collection Process

An electronic template spreadsheet (Microsoft Excel, Microsoft Corporation, Redmond, WA, USA) was developed to guide data abstraction for each study that fulfilled inclusion in the review. The data abstraction was completed independently. The completed individual templates were then collated into one master spreadsheet, and the results were discussed. The last author reviewed the summary of outcomes, and any discrepancies were resolved by consensus.

Data Items 

Abstracted data included general information about the study, such as author, year of study, purpose of study, study design, sample size, and subject demographics. Additionally, key variables for the review included (1) the determination of skin color/level of pigmentation, or race/ethnicity, (2) the measure of hypoxia; and (3) other clinical features of the population.

Synthesis

The studies were grouped by the statistical outcome relating to the device bias: mean difference, prevalence, and odds. The first author synthesized data and wrote the initial results. The final draft was reviewed and edited by each co-investigator, and finally, the last author revised, added to, and finalized the manuscript. Any discrepancies that emerged during the revision process were resolved by the consensus of the first and last authors.

Results

In eight of the 10 reviewed studies, statistically significant differences were found between pulse oximeter SpO_2_ readings vs. SaO_2_ values for patients of darker skin pigments compared to patients of lighter skin pigments if the patients were in a true hypoxic state [22-29]. This SpO_2_-SaO_2_ discrepancy, or skin color-related error (SCRE), and its risks were enumerated throughout the reviewed publications. Several studies attempted to quantify SCRE and noted that at all SaO_2_ values, the SpO_2_ readings in dark vs. light-skinned individuals were approximately 1% [22,26] to 1.57% higher [28]. The agreement between SpO_2_ measurements and SaO_2_ worsens with hypoxemia, with the SCRE increasing nearly linearly with decreasing SaO_2_ [26,28]. 

Individual devices have been evaluated by different investigators. Bickler et al. identified SCRE in three different pulse oximeters that overestimated SpO_2_ during hypoxia in dark-skinned patients: the Nellcor N-595 with the OxiMax-A probe, the Novametrix 513, and the Nonin Onyx [26]. The Nellcor device had an SCRE at all SaO_2_ ranges. At hypoxemic conditions, SCRE was found in the Novametrix device for SaO_2_ values between 60%-80%, and in the Nonin device only at an SaO_2_ of 70%-80%. For the 60%-70% SaO_2_ range, the SCRE between light and dark-skinned subjects for the Nonin device was +1.4%, which was the lowest of the group. The Nellcor and Novametrics devices had similar SCRE values at +4.3% and +4.4%, respectively. In regression analysis, the difference in slopes between light and dark skin was greatest with the Nellcor device and least with the Nonin device [26].

In patients admitted for COVID-19 with an SaO_2_ range of 85%-95%, Crooks et al. observed that pulse oximetry overestimated SpO_2_ in Black (+5.4%), Asian (+5.1%), or mixed-ethnicity (+6.9%) patients compared to White (+3.2%) patients [27]. The final mixed-effects model indicated that pulse oximetry overestimated SaO_2_ by a mean of +1.4% in Black, Asian, or mixed-ethnicity patients compared to White patients. Burnett et al. found that the overall SpO_2_ device bias for all ethnicities was 0.0±6.8%. But when stratified by race/ethnicity, White patients had a negative bias (-0.2±6.3%), while other groups had positive biases: Black (0.6±9.1%), Hispanic (0.5±7.9%), Asian (0.2±6.5%), and other (0.1±5.9%) [23].

Three studies found divergent results on the effect of skin pigmentation on SpO_2_ measurements [24,28,30]. Foglia et al. saw no significant difference in SCRE for the Masimo (0.8±4.2%) and the Nellcor (3.9±5.0%) in infants who had SaO_2_ values between 60%-92%; however, the SCRE between the two devices was significant [30]. According to Henry et al., there was no difference in estimated SaO_2_ values between Black patients (-0.08% [-0.27-0.11%]; p=0.392) and White patients, but they did identify a difference in Asian (-0.43% [-0.70 to -0.15%]; p=0.002) and American Indian (-0.68% [-0.96 to -0.40%]; p<0.001) patients compared to White patients [24]. In Smith and Hofmeyr’s comparison of perioperative patients in normal (SpO_2_ ≥93%) and “hypoxemic” groups (SpO_2_ <93%), the SCRE for normal (-0.20%) fell into the limits of agreement (LOA; -2.20 to -2.27%), while the SCRE for the hypoxic group fell outside of the LOA [28]. While they did not observe any trend indicating skin tones adversely affect SpO_2_ readings, they did find that generally, at hypoxic levels, they displayed worsening agreement. However, the study did not have enough statistical power to detect differences among skin tones [28].

The effect of SCREs is further demonstrated by the findings of Wong et al., who reported major differences in the accuracy of SpO_2_ readings across racial and ethnic groups, with the highest rate of “hidden (occult) hypoxemia” found in Black populations (6.9%), followed by Hispanics (6.0%), and less in Asians (4.9%) and Whites (4.9%). These higher rates of occult hypoxemia were associated with higher rates of subsequent organ dysfunction and in-hospital mortality, leading to poorer outcomes [29]. Burnett et al. also found a significantly greater prevalence of occult hypoxia in Black (2.1%) and Hispanic patients (1.8%) than in White patients (1.1%) at a SpO_2_ range of 92%-100%. To achieve a risk of less than 10% occult hypoxemia, Wong et al. described the following SpO_2_ thresholds: > 93% for Asians, > 96% for Black patients, > 92% for Hispanics, and > 93% for White patients [29]. Burnett et al. determined that the optimal SpO_2_ thresholds for predicting occult hypoxemia were > 94% for White patients, > 96% for Black patients, and > 95% for Hispanic patients [23]. These thresholds may be interpreted as follows. To be reasonably confident that occult hypoxemia is not present in a patient with the given ethnicity, their SpO_2_ value should be at or greater than the threshold.

The results of Chesley et al. are consistent with the findings that occult hypoxemia is most common among Black patients (7.9%) but also more common in all minority groups (ranging from 1.5%-4.8%) compared to White patients (2.9%). Additionally, they found a large variation in SCRE, with the highest intra-subject standard deviation of SCRE in Black subjects [25]. Interestingly, Seitz et al. observed that Black patients, compared to White patients, are more likely to have both hypoxemia (3.5% in Black patients, 1.1% in White patients) and hyperoxemia (4.7% in Black patients, 2.4% in White patients) at a SpO_2_ range of 92%-95% [22].

During hospitalization, Henry et al. determined that the estimated probability of occult hypoxemia in Black patients was 6.2% (5.1%-7.6%), in Asian patients, it was 6.6% (4.9%-8.8%), and in American Indian patients, it was 6.6% (4.4%-10.0%). [24]. Contrastingly, in White patients, it was 3.6% (3.4%-3.8%). At an SpO_2_ of 92%-95%, Black (7.6%), Asian (6.9%), and American Indian (7.3%) patients had a higher likelihood of experiencing occult hypoxemia compared to White patients (4.7%) [24]. The probability of hypoxemia (SaO_2_ <88%) increases with decreasing SpO_2_, with Black (36.0%), Asian (32.8%), and American Indian (39.3%) patients experiencing occult hypoxemia at a SpO_2_ of 88%-91%. After multivariable adjustment, Black patients were 65% more likely to have occult hypoxemia compared to White patients (odds ratio (OR) 1.65 [1.28-2.14]; p < 0.001) [24]. Occult hypoxemia was associated with increased odds of hospital mortality in surgical (OR 2.96 [1.20-7.28]; p=0.019) and ICU patients (OR 1.3 [1.03-1.08]; p=0.033) [24]. Burnett et al. calculated similar odds ratios for Black patients (OR 1.44 [1.11-1.87]) and Hispanic patients (OR 1.31 [1.03-1.68] compared to White patients [23].

Discussion

Results from most studies reviewed indicate that darker skin pigmentation is associated with a greater likelihood of SCRE in SpO_2_ values, with an error that increases if SaO_2_ values decrease to hypoxemic levels. In addition, COVID-19 patients from Asia and those who identified as Black had more unfavorable outcomes from the infection and were also more likely to have their SaO_2_ values overestimated based on pulse oximeter measurements [27]. The timely and accurate detection of oxygen saturation status using peripheral SpO_2_ measurements assumes even greater importance given the reported increased mortality rates found in critically ill patients [24]. The overall findings of this review strongly suggest that greater effort should be mustered to design and standardize such devices to minimize the SCRE and thereby reduce the potential negative health impacts on this population.

Barker et al. examined the Masimo oximeter and compared SpO_2_ and SaO_2_ measurements in healthy Black and White patients. The statistical bias (mean difference) and precision (standard deviation) values were -0.2 ± 1.40% for Black patients and -0.05 ± 1.35% for White patients, which was significant (p<0.001). However, it is notable that in this study, occult hypoxemia occurred in 0% of Black subjects and 0.2% of White subjects [31]. While the effect of SCREs may still be present in this device, the ability to prevent occult hypoxemia and improve clinical outcomes is a valuable progression. Additionally, Elron et al. examined the SpO_2_-SaO_2_ differences in children with cyanotic congenital heart disease using two pulse oximetry techniques: emitted red and infrared light (R&IR) and light with two wavelengths in the infrared region (2IR). The study concluded that 2IR better estimated SaO_2_ than R&IR techniques based on a lower standard deviation (3.6% and 6.5%, respectively) and higher correlation coefficients (0.94 and 0.83, respectively) [32]. Further device head-to-head comparison studies of this technology, accounting for skin color, are needed.

Strengths and limitations

This scoping review assessed publications across the CINAHL, PubMed, and Web of Science databases, allowing for a comprehensive search of existing literature. Only prospective and retrospective studies meeting our search term criteria were included; therefore, relevant research in other study designs may have been excluded. The search terms used may have also left out publications using different terminology to describe hypoxia. Some studies we analyzed lacked specific race and ethnicity data (i.e., using light skin vs. dark skin comparisons), and most studies used self-reported race to categorize patients [22-25]. There can be a wide range of skin tones within racial and ethnic groups, which is not always accounted for. A more direct method of measuring skin pigmentation would have been more accurate. Small sample sizes and a lack of racial diversity were also limitations in several studies [26,30]. Most studies focused on adult populations and did not account for underlying diseases that might affect SpO_2_ readings [22-28]. In addition, different oximetry devices showed discrepancies in the accuracy of their readings, which should be considered, especially in hypoxic or near-hypoxic conditions [26,30].

Future implications

In the future, studies should have: 1) more systematic measurement of skin tones; 2) more studies should explore the negative effects of skin tone in pediatric monitoring; and 3) studies should have larger sample sizes.

## Conclusions

This scoping review found that non-White patients had a higher probability of occult hypoxemia than their White counterparts. Patients with darker complexions had overestimated SpO_2_ values during hypoxia compared to patients with lighter complexions. This evidence indicates that skin color impacts the accuracy of the SpO2, particularly when the SaO_2_ values are in a hypoxemic range. The potential implications of these faulty readings are broad. Firstly, clinical decisions heavily rely on SpO_2_, especially in the wake of the COVID-19 pandemic. Undetected hypoxemia is associated with adverse health outcomes in patients, including increased in-hospital mortality and complications such as organ dysfunction. Subsequently, overestimating SpO_2_ can delay the delivery of life-saving treatment to critically ill patients requiring respiratory rehabilitation and supplemental oxygen therapy. This review indicates that more research is needed to improve pulse oximeter models to better characterize skin tones susceptible to the discovered errors in SpO_2_ values. Biomedical sensors that are inclusive of all pigmentations will optimize individual healthcare statuses, minimize disparities in treatment, and decrease mortality.
